# Serum 25-hydroxyvitamin D_3_ and body composition in an elderly cohort from Germany: a cross-sectional study

**DOI:** 10.1186/1743-7075-9-42

**Published:** 2012-05-18

**Authors:** Alexandra Jungert, Heinz J Roth, Monika Neuhäuser-Berthold

**Affiliations:** 1 Institute of Nutritional Science, Justus-Liebig-University, Goethestrasse 55, 35390, Giessen, Germany; 2 Endocrinology & Oncology Department, Limbach Laboratory, 69126, Heidelberg, Germany

**Keywords:** 25-Hydroxyvitamin D_3_, Body composition, Fat mass, Elderly

## Abstract

**Background:**

Emerging evidence indicates that there is an association between vitamin D and obesity. The aim of this study was to investigate whether the level of serum 25-hydroxyvitamin D_3_ [25(OH)D_3_] in the elderly is influenced by parameters of anthropometry and body composition independent of potential confounding lifestyle factors and the level of serum intact parathyroid hormone (iPTH).

**Methods:**

Cross-sectional data of 131 independently living participants (90 women, 41 men; aged 66–96 years) of the longitudinal study on nutrition and health status in senior citizens of Giessen, Germany were analysed. Concentrations of 25(OH)D_3_ and iPTH were ascertained by an electrochemiluminescence immunoassay. Body composition was measured by a bioelectrical impedance analysis. We performed univariate and multiple regression analyses to examine the influence of body composition on 25(OH)D_3_ with adjustments for age, iPTH and lifestyle factors.

**Results:**

In univariate regression analyses, 25(OH)D_3_ was associated with body mass index (BMI), hip circumference and total body fat (TBF) in women, but not in men. Using multiple regression analyses, TBF was shown to be a negative predictor of 25(OH)D_3_ levels in women even after controlling for age, lifestyle and iPTH (*ß* = −0.247; *P* = 0.016), whereas the associations between BMI, hip circumference and 25(OH)D_3_ lost statistical significance after adjusting for iPTH. In men, 25(OH)D_3_ was not affected by anthropometric or body composition variables.

**Conclusions:**

The results indicate that 25(OH)D_3_ levels are affected by TBF, especially in elderly women, independent of lifestyle factors and iPTH.

## Background

There is emerging evidence that, in addition to its well-established role in the regulation of calcium homeostasis and bone metabolism, vitamin D has multiple functions in human health. Accumulating epidemiological data indicate that a low vitamin D status is linked to a variety of chronic diseases that are associated with aging, including cancer, autoimmune diseases, hypertension and diabetes mellitus [1-3]. Elderly people are especially at risk for a vitamin D deficiency because of age-related declines in the endogenous vitamin D synthesis, sun exposure and dietary intake [4]. It is a moot point whether adiposity, which is increasingly prevalent in people with advanced age [5], may also negatively impact the vitamin D status in the elderly. Numerous studies have linked low 25-hydroxyvitamin D levels [25(OH)D], the commonly used indicator of the vitamin D status [6], with obesity [7-21], whereas other researchers have failed to confirm this observation [22-24]. There are some limitations of these previous studies. First, these studies often included only women, vitamin D-deficient individuals, ambulatory patients, morbidly obese subjects or subjects of young or middle age, and the interpretation of these results is limited, especially in reference to elderly individuals. Second, the majority of these studies relied on body mass index (BMI) without a further ascertainment of total body fat (TBF). Third, some of these studies did not control for potential confounders, such as age, nutrient intake, use of vitamin D supplements, daily sun exposure, physical activity and smoking, which may be associated with both vitamin D status and adiposity. In this context, intact parathyroid hormone (iPTH) is also expected to be a relevant confounder or even the causal factor of the association between TBF and 25(OH)D [25], and previous studies often concentrated exclusively on 25(OH)D or iPTH without considering the interaction between both. Fourth, anthropometric and body composition variables were frequently included simultaneously in the multiple regression model. Consequently, these results must be interpreted with caution.

Overall, studies in which the associations of anthropometry and body composition with 25(OH)D were examined in predominantly non-obese, well-functioning elderly women and men are scarce. Therefore, and in view of the inconsistent results and the above mentioned limitations of previous studies, the primary objective of our study was to analyse whether anthropometric characteristics and body composition contribute to the 25-hydroxyvitamin D_3_ [25(OH)D_3_] status in non-institutionalised, primarily non-obese elderly women and men. In this context, potential confounders of this association, such as age, sun exposure, physical activity, vitamin D and calcium intake, smoking history, alcohol consumption and iPTH, were considered. In this way, we could scrutinise whether anthropometric and body composition variables are independent predictors for 25(OH)D_3_ levels in the elderly or whether age, lifestyle or iPTH may account for the effects of body composition on the 25(OH)D_3_ status.

## Methods

### Subjects

Subjects were participants of the GISELA study, a prospective cohort study in which the nutrition and health status of senior citizens from Giessen (50°35′North), Germany, have been observed since 1994. All investigations took place in the Institute of Nutritional Science in Giessen from July to October. For enrolment, subjects had to be at least 60 years of age and able to visit the institute without assistance. A written informed consent was obtained from each participant. The study protocol was approved by the Ethical Committee of the Faculty of Medicine at the Justus-Liebig-University, Giessen.

The present investigation reports cross-sectional data from the GISELA study obtained in 2008. Subjects with incomplete data were excluded. Those individuals who took diuretics, had undergone a hormone replacement therapy or suffered from chronic kidney disease or oedema were also excluded from the analysis. Eight people were characterised as outliers due to their serum 25(OH)D_3_ and iPTH measurements as well as the residuals of the regression analyses and were therefore not included. Of the 275 elderly people who took part in the follow-up in 2008, data from 90 women and 41 men were included in the following analysis.

### Anthropometric data and body composition

Body weight was determined by a calibrated digital scale (Seca, Vogel & Halke, Hamburg, Germany) to the nearest 0.1 kg in light clothes without shoes. Based on the weight of the remaining clothes, 0.5 to 1.0 kg was subtracted from the measured weight. Body height was recorded standing upright without shoes via a telescopic rod integrated in the scale to the nearest 0.5 cm. The BMI categories were established such that subjects with a BMI < 25.0 kg/m² were classified as normal weight, whereas participants with a BMI ≥ 25.0 kg/m² were defined as overweight to obese. The waist-to-hip ratio (WHR) and waist circumference (WC) were used as markers for body fat distribution. Abdominal obesity was defined according to the WHO [26] as a WHR > 0.85 for women and > 1.0 for men or a WC ≥ 88 cm for women and ≥ 102 cm for men. The WC and hip circumference (HC) were determined in an upright position by a tape measure to the nearest 1.0 cm.

Body composition was recorded by a single-frequency (50 kHz) bioelectrical impedance analyser (Akern-RJL BIA 101/S, Data Input, Frankfurt, Germany) according to the instructions of the manufacturer and the predictive formula from Roubenoff et al. [27].

### Laboratory measurements

Blood samples for serum 25(OH)D_3_ and iPTH were collected between 7:00 a.m. and 11:00 a.m. after an overnight fast and serum aliquots were stored at −70°C until further analysis. Both 25(OH)D_3_ and iPTH were measured by a direct electrochemiluminescence immunoassay (ECLIA, Roche Diagnostics, Mannheim, Germany) [28,29]. Importantly, the ECLIA specifically detects the 25(OH)D_3_ concentration. We defined 25(OH)D_3_ < 25.0 nmol/L as vitamin D-deficient, 25.0–49.9 nmol/L as insufficient and ≥ 50.0 nmol/L as adequate. In addition, we accounted for the ongoing debate concerning the use of higher cut-off values by means of an accessory cut-off value of ≥ 75.0 nmol/L [1,30].

### Lifestyle factors

A three-day estimated dietary record, which was developed and validated for the GISELA study, functions to determine the nutritional intake of each subject [31]. Smoking behaviour, current daily time spent outdoors, physical activity pattern and further data, such as age, diseases, medications and vitamin D supplement intake, were collected using self-administered questionnaires. The current time spent outdoors (min/d) was used as an indicator for sun exposure. The smoking behaviour (never-smokers vs. current and ex-smokers) and the use of vitamin D supplements (no/yes) were coded as dichotomous variables. The physical activity level (PAL) of each participant was assessed as described elsewhere [32].

### Statistical analysis

The characteristics of the study subjects were expressed as medians and the 5^th^–95^th^ percentiles due to non-normally distributed data. Because of significant sex differences in the amount of TBF, fat distribution and lifestyle factors, we performed sex-stratified analyses. According to the sample size, a normal distribution was tested by the Shapiro-Wilk test for men and by the Kolmogorov-Smirnov test with the Lilliefors correction for women. Descriptive characteristics were compared between groups via the Mann-Whitney *U* test for continuous variables and via the χ² test or, alternatively, Fisher’s exact test for categorical variables.

Whenever subjects with an adequate vitamin D status were compared with vitamin D-insufficient individuals, we used two different 25(OH)D_3_ cut-off values (≥ 50 nmol/L and ≥ 75 nmol/L). The study cohort was split by BMI into two groups to compare normal-weight subjects with overweight to obese participants for the prevalence of vitamin D insufficiency by means of the Fisher’s exact test. In addition, we divided the cohort into subjects with abdominal obesity and participants without an abdominal fat distribution, as defined by the WHR and WC, and analysed whether differences concerning the prevalence of vitamin D insufficiency exist. Furthermore, subjects with an adequate vitamin D status were compared with vitamin D-insufficient individuals with regard to parameters of anthropometry and body composition by using the Mann-Whitney *U* test.

We examined univariate associations of 25(OH)D_3_ with iPTH and parameters of lifestyle, anthropometry and body composition by simple regression analyses. Serum iPTH was regarded as a covariate because iPTH can promote the turnover of circulating 25(OH)D_3_ by inducing the formation of 1,25-dihydroxyvitamin D_3 _[33]. On the basis of non-normally distributed residuals, the dependent variable 25(OH)D_3_ was logarithmically transformed (log_10_) to obtain a normal distribution of residuals.

Finally, we performed sex-stratified hierarchical multiple regression analyses. In detail, we created three models with different levels of adjustment. In each of the three models, the log 25(OH)D_3_ was considered as the dependent variable and either the WC, HC, WHR, BMI or % TBF was included as a predictor variable. The fat-free mass (FFM), body weight and absolute TBF were not tested either because of their limited or somewhat lower explanatory power compared with the other anthropometric variables or the % TBF, and therefore were not expected to provide any additional information. Model 1 represents each association of the anthropometric and body composition variables with the log 25(OH)D_3_ adjusted for age, while model 2 additionally adjusts for lifestyle factors (PAL, sun exposure, smoking, use of vitamin D supplements, alcohol consumption, vitamin D and calcium intake). Model 3 comprises all covariates of model 2 and additionally considers iPTH as a confounding variable. Statistical analyses were done using the SPSS 18.0 statistical package for Windows (SPSS Inc., Chicago, USA). The significance level was set at *P* < 0.05. All tests were two-tailed.

## Results

### Characteristics of the study subjects

The characteristics of the subjects are presented in Table 1. None of the subjects had a serious vitamin D deficiency, but 25.6 % of the women and 19.5 % of the men had 25(OH)D_3_ levels < 50 nmol/L. Levels ≥ 75 nmol/L were observed in 18.9 % of the females and 22.0 % of the males.

**Table 1:** Descriptive characteristics of the study population

**Characteristics**	**Women (*n*** **= 90) Median (P**_**5**_**–P**_**95**_**)**	**Men (*n*** **= 41) Median (P**_**5**_**–P**_**95**_**)**	***P*-value **^**a)**^
Age (y)	75.5 (69.0–86.5)	76.0 (70.0–84.8)	0.546
Height (cm)	160.0 (150.5–168.7)	174.5 (161.6–183.5)	< 0.0001
Weight (kg)	67.8 (52.9–85.2)	78.5 (63.7–98.0)	< 0.0001
Body mass index (kg/m²)	26.3 (20.8–34.5)	26.4 (22.9–32.3)	0.806
Waist circumference (cm)	90.0 (71.6–108.4)	100.0 (84.4–113.8)	< 0.0001
Hip circumference (cm)	106.0 (91.6–122.5)	104.0 (95.2–116.8)	0.170
Waist-to-hip ratio	0.85 (0.76–0.93)	0.96 (0.86–1.06)	< 0.0001
Fat-free mass (kg)	39.5 (34.5–45.1)	55.3 (48.6–62.6)	< 0.0001
Total body fat (kg)	28.1 (17.2–41.6)	24.2 (14.4–36.0)	0.001
Total body fat (%)	41.9 (32.4–50.1)	29.0 (21.5–37.9)	< 0.0001
25(OH)D_3_ (nmol/L)	59.4 (39.9–90.6)	67.5 (39.7–88.9)	0.096
iPTH (pmol/L)	4.5 (2.3–7.9)	4.1 (2.1–8.3)	0.360
Vitamin D intake (μg/d)	2.3 (0.3–10.1)	3.5 (1.0–11.3)	0.078
Calcium intake (g/d)	1.0 (0.5–1.8)	1.0 (0.6–1.6)	0.953
Alcohol intake (g/d)	2.3 (0.0–16.1)	5.1 (0.0–30.0)	0.018
Sun exposure (min/d)	120.0 (40.0–360.0)	150.0 (22.5–396.0)	0.130
Physical activity level	1.7 (1.5–2.0)	1.7 (1.4–1.9)	0.254
Current or ex-smokers, n (%)	20 (22.2)	29 (70.7)	< 0.0001
Vitamin D supplement users, n (%)	15 (16.7)	2 (4.9)	0.091

Abbreviations: 25(OH)D_3_, 25-hydroxyvitamin D_3_; iPTH, intact parathyroid hormone.

^a)^ Tests of significance between the sexes were based on the Mann-Whitney *U* test for continuous variables and the χ² test for categorical variables.

### Adiposity and the prevalence of vitamin D insufficiency by using two cut-off values

The women with BMIs ≥ 25 kg/m² had a higher prevalence of vitamin D insufficiency compared to the normal-weight women when the cut-off value of 75 nmol/L was applied (69.9 % vs. 30.1 %; *P* = 0.012), but not when the lower value of 50 nmol/L was used (*P* = 0.132). Men showed no differences regarding the prevalence of vitamin D insufficiency when stratified according to BMI, which was independent of the cut-off value that was used (both *P* > 0.200). No differences were found after dividing the cohort into subjects with abdominal obesity and those without, independent of sex (all *P* > 0.200). In women, but not in men, the BMI, WC and HC were higher in subjects with 25(OH)D_3_ levels < 50 nmol/L compared to subjects with 25(OH)D_3_ levels ≥ 50 nmol/L (all *P* < 0.05). When using the cut-off value of 75 nmol/L, the % TBF was significantly higher in both the female and male subjects with 25(OH)D_3_ levels < 75 nmol/L (both *P* < 0.05).

### Univariate associations between 25-hydroxyvitamin D_3_ and other parameters

**Figure 1:**
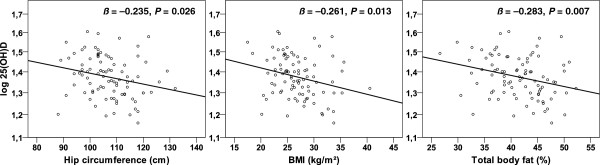
**Association of body composition with the log-transformed 25-hydroxyvitamin D**_**3**_**in elderly women.** Associations between serum 25-hydroxyvitamin D_3_ (25(OH)D_3_) and the hip circumference (left panel), BMI (middle panel) and percentage of total body fat (right panel) in women aged 66 to 96 years (*n* = 90). The *P* values and the standardised coefficients *ß* were calculated by univariate regression analyses.

Table 2 provides the results of the univariate linear regression analyses. In women, the log 25(OH)D_3_ was associated with body weight, HC, BMI, absolute and % TBF, alcohol consumption, sun exposure, PAL and iPTH. In men, sun exposure, PAL and current or past smoking significantly affected the log 25(OH)D_3_. All other parameters, including age, WC, WHR, absolute FFM and intake of vitamin D, calcium and vitamin D supplements, were not associated with the log 25(OH)D_3_. The linear relationships of the log 25(OH)D_3_ with HC, BMI and % TBF in women are illustrated in Figure 1.

**Table 2:** **Univariate linear regression analyses between the log-transformed 25-hydroxyvitamin D**_**3**_**and other parameters**

**Characteristics**	**log 25(OH)D**_**3**_			
	**Women (*n*** **= 90)**		**Men (*n*** **= 41)**	
	***ß***	***P*-value**	***ß***	***P*-value**
iPTH (pmol/L)	−0.379	< 0.001	−0.212	0.182
Age (y)	−0.107	0.315	−0.200	0.209
Weight (kg)	−0.240	0.023	0.103	0.521
Body mass index (kg/m²)	−0.261	0.013	−0.040	0.806
Waist circumference (cm)	−0.181	0.088	−0.126	0.434
Hip circumference (cm)	−0.235	0.026	−0.039	0.809
Waist-to-hip ratio	−0.029	0.789	−0.140	0.382
Fat-free mass (kg)	−0.137	0.199	0.268	0.090
Total body fat (kg)	−0.261	0.013	−0.012	0.939
Total body fat (%)	−0.283	0.007	−0.088	0.584
Vitamin D intake (μg/d)	−0.029	0.788	−0.092	0.568
Calcium intake (g/d)	−0.049	0.645	−0.015	0.925
Alcohol intake (g/d)	0.211	0.046	0.100	0.533
Sun exposure (min/d)	0.301	0.004	0.370	0.017
Physical activity level	0.212	0.044	0.442	0.004
Current or past smoking ^a)^	0.007	0.946	−0.520	< 0.001
Vitamin D supplement use ^a)^	0.134	0.207	0.172	0.283

Abbreviations: log 25(OH)D_3_, log-transformed 25-hydroxyvitamin D_3_; *ß*, standardised coefficient; iPTH, intact parathyroid hormone.

^a)^ Dummy variable (no/yes).

### Adjusted associations of 25-hydroxyvitamin D_3_ with anthropometric and body composition variables

The results of the multiple regression analyses with different levels of adjustment are shown in Table 3. Due to the observed collinearity of the anthropometric and body composition variables (data not shown), these variables were added as potential predictor variables in separate models by replacing each other. No associations existed between the log 25(OH)D_3_ and WC or WHR in either sex. In men, the BMI, HC and % TBF were not associated with the log 25(OH)D_3_, independent of the level of adjustment. In contrast, the % TBF emerged as a negative predictor for the log 25(OH)D_3_ in women both before and after an additional adjustment for iPTH, whereas the BMI and HC were not significantly associated with the log 25(OH)D_3_ after a full adjustment that included iPTH. In women, the fully adjusted model 3, with % TBF as a predictor variable, explained 27.4 % of the variation in 25(OH)D_3_ levels and the % TBF accounted for 4.9 % of the variation in this regard. Besides the % TBF, iPTH (*ß* = −0.345; *P* < 0.001) and sun exposure (*ß* = 0.239; *P* = 0.018) were independent determinants of the log 25(OH)D_3_ in women, while alcohol intake showed a borderline significant association (*ß* = 0.188; *P* = 0.052). In men, only smoking (*ß* = −0.438; *P* = 0.005) had an independent impact on the log 25(OH)D_3_. When we created multiple regression models including only those variables as confounders that exhibited a significant association with the log 25(OH)D_3_ in the univariate analyses, so that iPTH levels, alcohol intake, sun exposure and PAL were included in the female model and sun exposure, PAL and smoking behaviour in the male model, this yielded equal results (data not shown).

**Table 3:** **Multiple regression analyses between 25-hydroxyvitamin D**_**3**_**and the respective anthropometric or body composition parameter**^**a)**^

	**Women (*n*** **= 90)**			**Men (*n*** **= 41)**		
	**log 25(OH)D**_**3**_			**log 25(OH)D**_**3**_		
	**B**	***ß***	***P*-value**	**B**	***ß***	***P*-value**
**WC (cm)**						
Model 1	−0.002	−0.189	0.075	−0.001	−0.146	0.362
Model 2	−0.002	−0.176	0.096	−0.001	−0.097	0.489
Model 3 ^b)^	−0.001	−0.155	0.115	−0.001	−0.071	0.632
**HC (cm)**						
Model 1	−0.003	−0.260	0.015	−0.001	−0.043	0.786
Model 2	−0.002	−0.202	0.058	−0.001	−0.042	0.769
Model 3 ^b)^	−0.002	−0.141	0.161	−0.0004	−0.026	0.855
**WHR**						
Model 1	−0.035	−0.018	0.869	−0.252	−0.168	0.293
Model 2	−0.137	−0.070	0.522	−0.157	−0.105	0.462
Model 3 ^b)^	−0.251	−0.128	0.208	−0.118	−0.079	0.602
**BMI (kg/m²)**						
Model 1	−0.007	−0.285	0.007	−0.003	−0.084	0.608
Model 2	−0.006	−0.240	0.026	−0.002	−0.060	0.678
Model 3 ^b)^	−0.005	−0.186	0.065	−0.001	−0.037	0.803
**TBF (%)**						
Model 1	−0.006	−0.315	0.003	−0.002	−0.104	0.517
Model 2	−0.005	−0.284	0.010	−0.001	−0.031	0.827
Model 3 ^b)^	−0.005	−0.247	0.016	−0.0003	−0.014	0.920

Abbreviations: WC, waist circumference; HC, hip circumference; WHR, waist-to-hip ratio; BMI, body mass index; TBF, total body fat.

^a)^ Multiple regression analyses using the log-transformed 25-hydroxyvitamin D_3_ as the dependent variable and the respective anthropometric and body composition parameters as the predictor variables. The results of the regression analyses are expressed in terms of B (the unstandardised coefficient), *ß* (standardised coefficient), and the adjusted coefficient of determination (R^2^) for the final model 3. Model 1: association adjusted for age (y). Model 2: model 1 with additional adjustments for physical activity level, sun exposure (min/d), vitamin D intake (μg/d), calcium intake (g/d), alcohol consumption (g/d), smoking (never-smokers *vs*. current or ex-smokers) and use of vitamin D supplements (no/yes). Model 3: model 2 controlled for intact parathyroid hormone (pmol/L).

^b)^ Adjusted coefficient of determination (R^2^) for the respective model 3 (including the respective anthropometric and body composition variable and all covariates) for women (WC = 0.243; HC = 0.238; WHR = 0.235; BMI = 0.252; and % TBF = 0.274) and for men (WC = 0.275; HC = 0.270; WHR = 0.276; BMI = 0.271; and % TBF = 0.270).

## Discussion

To our knowledge, this is the first study that examines the associations of anthropometric and body composition variables with the vitamin D status of predominantly non-obese, non-vitamin D-deficient, well-functioning elderly women and men from Germany while also considering the potentially confounding effects of age, lifestyle factors and iPTH.

In contrast to other studies of elderly people [15,23,34], none of the GISELA subjects had a severe vitamin D deficiency. Moreover, while in our investigation fewer than 25 % of the subjects had 25(OH)D_3_ levels < 50 nmol/L, the prevalence of vitamin D insufficiency in the general German population aged 65–79 years exceeds 50 %, even during the summer [34].

Previous studies have reported a higher prevalence of vitamin D insufficiency in obese subjects compared to lean individuals [9,35]. In our study, the prevalence of vitamin D insufficiency was 39.8 % lower in normal-weight women than in women with BMIs ≥ 25 kg/m² when using the cut-off value of 75 nmol/L. When our study cohort was stratified by 25(OH)D_3_ status, individuals with an adequate vitamin D status (≥ 75 nmol/L) had a lower % TBF than subjects with 25(OH)D_3_ levels < 75 nmol/L. This suggests that a higher % TBF may have limited the increase in 25(OH)D_3_ levels over the threshold of 75 nmol/L or, alternatively, a sufficient vitamin D status may have protected against an increasing TBF.

At present there is a great debate on the 25(OH)D_3_ target thresholds as regards multiple health outcomes and hence guidelines for dietary intakes. While the Institute of Medicine (IoM) suggests 50 nmol/L as the target value for dietary reference intakes of vitamin D [36], others favour 75 nmol/L or even higher values as sufficiency threshold [30]. We observed distinct differences in the % TBF of subjects with vitamin D insufficiency only when using 75 nmol/L as cut-off level, whereas anthropometric variables already differed between subjects with 25(OH)D_3_ levels ≥ 50 nmol/L and those with < 50 nmol/L. As the multiple regression analyses revealed that the % TBF, but none of the anthropometric variables, is a key determinant of 25(OH)D_3_ levels, this might favour the view that the threshold of 75 nmol/L reflects a more adequate cut off-value than 50 nmol/L. However, the observed linear relationship between % TBF and 25(OH)D_3_ indicates that a defined threshold concentration of 25(OH)D_3_ with regard to adiposity may not exist in our study population of non-vitamin D-deficient elderly individuals. Consequently, the requirements for vitamin D of individuals to achieve or exceed a defined cut-off level may depend on their % TBF, which may be highly variable even at a given BMI.

In our univariate analyses, the anthropometric and body composition variables had an impact on 25(OH)D_3_ only in women, which may be attributed to the small sample size of men and to the lower % TBF in men and the unequal fat distribution between the female and male subjects. Of the anthropometric and body composition variables examined in our study, the % TBF was the strongest predictor of the 25(OH)D_3_ status in the women. An inverse association between the concentration of 25(OH)D and % TBF was also reported in a study of 410 women aged 20–80 years [8] and in a study of 112 postmenopausal women [18]; however, no association of 25(OH)D levels with BMI was found in either study. In contrast, 25(OH)D levels were negatively affected by the BMI, but not by the % TBF, in a study of only overweight and obese middle-aged subjects [37]. Other authors [20] reported an inverse association of 25(OH)D levels with the % TBF in elderly people, which was stronger in women and stronger than the associations of 25(OH)D levels with the anthropometric variables. Our results support the conclusion that the % TBF represents a more potent predictor of the 25(OH)D_3_ status compared with BMI or other anthropometric variables.

We did not find a relationship of 25(OH)D_3_ levels with the WC or WHR, which does not support the hypothesis that the abdominal fat tissue exerts an important effect on the vitamin D status, as others have suggested [12-14,38]. Snijder et al. [20] reported that a greater WC had a negative impact on 25(OH)D levels in both sexes, whereas the WHR was not associated with 25(OH)D levels. Moschonis et al. [18] observed no associations of 25(OH)D levels with either the WC or HC in postmenopausal women. In our study, the negative impact of the HC on 25(OH)D_3_ levels indicates a specific role of peripheral or subcutaneous fat tissue, which is in line with the hypothesis that a low 25(OH)D_3_ status is a consequence of an increased sequestration of vitamin D in adipose tissue, especially in the subcutaneous fat [39].

At present, it is unclear whether adipose tissue acts as a reservoir for vitamin D by releasing vitamin D into the circulation when required or as a metabolic trap that reduces its bioavailability [6,39]. Moreover, there is uncertainty about the causal character of the association between TBF and 25(OH)D. In this context, it has been hypothesised that a low 25(OH)D status may contribute to obesity by promoting secondary hyperparathyroidism [25,40]. Alternatively, it has been postulated that iPTH may promote weight gain independent of vitamin D by possibly promoting lipogenesis and inhibiting lipolysis [25,40]. We observed an inverse association of 25(OH)D_3_ with iPTH in women, as reported by others [7,18,33]. In men, we did not find this association, which may be due to hormonal differences and the sample size. Although the association between the % TBF and 25(OH)D_3_ diminished in our study after adjusting for iPTH, the association remained statistically significant. Thus, iPTH does not appear to be responsible for the inverse association of the % TBF with 25(OH)D_3_ in our subjects, which is in line with previous studies [18,20]. Contrary to the % TBF, the associations between anthropometric variables and 25(OH)D_3_ in women were abolished after adjusting for iPTH, which may be because the BMI does not adequately reflect the % TBF. The marginal effect of iPTH on the association of % TBF with 25(OH)D_3_ in our study may possibly due to the relatively high vitamin D status of our participants.

Another suggestion is that differences in lifestyle may contribute to a lower vitamin D status in overweight individuals, such as decreased sun exposure because of clothing habits [41] or low exercise levels [42]. We noticed an association of sun exposure with 25(OH)D_3_, which is consistent with the literature [7,15,18], and an association between PAL and 25(OH)D_3_ in men, as also reported in other studies [10,17,19,35]. A low PAL may increase the TBF, which in turn may decrease the bioavailability of vitamin D. The physically inactive individuals also typically spend less time outdoors than the active individuals, which may lead to a lower cutaneous vitamin D production. While a positive association between the PAL and sun exposure was present in both sexes in our study, a significant association between the PAL and % TBF was only observed in women (data not shown). According to the results of the multiple regression analyses, the amounts of sun exposure and physical activity appear to not be responsible for the inverse association of the % TBF with 25(OH)D_3_, as also suggested by others [12,43]. Moreover, smoking, alcohol or the habitual intake of vitamin D, calcium or vitamin D supplements could not explain the association between the % TBF and vitamin D status in our study. Nevertheless, we noted an unexpected strong and independent negative association between smoking and 25(OH)D_3_ in men. While some previous studies reported a negative association [17,44,45], other researchers failed to confirm such a relationship [8,15]. Another finding of our study is the positive association between alcohol intake and 25(OH)D_3_ in women, which has also been reported by others [17,45]. Neither dietary nor supplemental vitamin D had an impact on 25(OH)D_3_ in our study, which is in contrast to some [15,17], but not all [8,11], previous studies. Given the low vitamin D intake from diet and supplements and the low percentage of supplement users in our study, these amounts may have been too low to affect 25(OH)D_3_ levels in non-vitamin D-deficient subjects.

Finally, we observed no age dependency of 25(OH)D_3_ levels although dermal vitamin D synthesis declines in advanced age [46]. It is possible that the age gradient was too small, but most likely, an age-related impairment of vitamin D synthesis is compensated for by adequate sun exposure in our subjects. This is in line with the observation that individuals aged ≥ 60 years are able to synthesise enough vitamin D during outdoor activities and thus show a 25(OH)D status similar to young adults [19]. However, we found that the association between the % TBF and 25(OH)D_3_ seems to strengthen after adjusting for age.

Our study has several strengths and weaknesses. For the strengths, this study examined both independently living elderly women and men without a vitamin D deficiency. A special feature in our approach was the consideration of age, iPTH and a variety of lifestyle factors as well as different cut-off levels of 25(OH)D_3_ that were used to evaluate the vitamin D status. In addition, confounding was minimised due to well-defined exclusion criteria. For the weaknesses, the cross-sectional design limited our ability to establish causal relationships. Due to the sample size, it is possible that some associations were classified as not statistically significant because of a type II error. Nevertheless, we found an independent and robust association between the % TBF and 25(OH)D_3_ levels in women. The subjects in this study were volunteers, had a higher educational level and were more aware of health issues than their peers in the general German population. In general, the study comparability is limited due to varying study designs among this and other studies, especially regarding the period of recruitment, assay methods and differences in the participants’ ages and BMIs. The use of different 25(OH)D assays has been a matter of dispute [47]. It is possible that the ECLIA used in this study has systematically over- or underestimated the 25(OH)D_3_ levels of our subjects. However, the measurement results of the ECLIA are in a good overall agreement with those determined by tandem mass spectrometry [48]. Further limitations are the use of self-reported data, the indirect measurements of sun exposure and physical activity and missing information on the exact dosage and duration of the intake of vitamin D supplements. Considering the seasonal variation of 25(OH)D_3_ levels, our data may not clearly reflect the year-long vitamin D status of our participants. Finally, while dual-energy X-ray absorptiometry (DXA) may reflect the body composition better than BIA, our BIA equation has been validated against the DXA. In general, BIA works well in subjects with a relatively stable water and electrolyte balance when using an appropriate and validated BIA equation [49].

## Conclusion

In conclusion, the present study provides evidence that, especially in elderly women, the % TBF can be an important negative determinant of the 25(OH)D_3_ status, which is for the most part independent of age, lifestyle factors and iPTH. Remarkably, we found this association during the summer in subjects who were primarily not obese and not vitamin D-deficient. Consequently, the requirements for vitamin D to achieve or exceed a defined cut-off level for 25(OH)D_3_ may depend on the % TBF, which may be highly variable even at a given BMI. Although our results indicate that, besides regular physical activity, adequate sun exposure and abstinence from smoking, a reduction in TBF may be one possible strategy to improve the vitamin D status, this may not easily be achievable or not even appropriate in the elderly. Therefore, and in view of the high prevalence of overweight and obesity and potential associations of vitamin D with a variety of chronic age-related diseases, a clinical implication might be that especially overweight, post-menopausal women should be screened for vitamin D deficiency and, as the case may be, specifically advised to increase their vitamin D intake either by supplements or vitamin D-enriched foods.

## Abbreviations

25(OH)D: 25-hydroxyvitamin D; 25(OH)D_3_: 25-hydroxyvitamin D_3_; iPTH: intact parathyroid hormone; GISELA: longitudinal study on nutrition and health status in senior citizens of Giessen, Germany: BMI: body mass index; WHR: waist-to-hip ratio; WC: waist circumference; HC: hip circumference; TBF: total body fat; FFM: fat-free mass; BIA: bioelectrical impedance analysis; ECLIA: electrochemiluminescence immunoassay; PAL: physical activity level; DXA: dual-energy X-ray absorptiometry; IoM: institute of Medicine.

## Competing interests

The authors declare that they have no competing interests.

## Authors’ contributions

AJ performed the statistical analysis, interpreted the data and wrote the manuscript. HJR analysed the blood samples. MNB designed the study, conducted the research and proofread the manuscript. All authors approved the final manuscript.

## References

[B1] Bischoff-Ferrari HA Giovannucci E Willett WC Dietrich T Dawson-Hughes B Estimation of optimal serum concentrations of 25-hydroxyvitamin D for multiple health outcomes Am J Clin Nutr 2006 84 18 28 16825677 10.1093/ajcn/84.1.18

[B2] Jungert A Roth HJ Neuhäuser-Berthold M Serum 25-hydroxyvitamin D3, parathyroid hormone and blood pressure in an elderly cohort from Germany: a cross-sectional study Nutr Metab (Lond) 2012 9 20 10.1186/1743-7075-9-20 22433818 PMC3362780

[B3] Alvarez JA Bush NC Choquette SS Hunter GR Darnell BE Oster RA Gower BA Vitamin D intake is associated with insulin sensitivity in African American, but not European American, women Nutr Metab (Lond) 2010 7 28 10.1186/1743-7075-7-28 20398267 PMC2868016

[B4] Hayes DP Vitamin D and ageing Biogerontology 2010 11 1 16 10.1007/s10522-009-9252-0 19844804

[B5] Houston DK Nicklas BJ Zizza CA Weighty concerns: the growing prevalence of obesity among older adults J Am Diet Assoc 2009 109 1886 1895 10.1016/j.jada.2009.08.014 19857630

[B6] Brannon PM Yetley EA Bailey RL Picciano MF Overview of the conference “Vitamin D and health in the 21st century: an update” Am J Clin Nutr 2008 88 483S 490S 18689388 10.1093/ajcn/88.2.483S

[B7] Ardawi MSM Qari MH Rouzi AA Maimani AA Raddadi RM Vitamin D status in relation to obesity, bone mineral density, bone turnover markers and vitamin D receptor genotypes in healthy Saudi pre- and postmenopausal women Osteoporos Int 2011 22 463 475 10.1007/s00198-010-1249-7 20431993

[B8] Arunabh S Pollack S Yeh J Aloia JF Body fat content and 25-hydroxyvitamin D levels in healthy women J Clin Endocrinol Metab 2003 88 157 161 10.1210/jc.2002-020978 12519845

[B9] Bischof MG Heinze G Vierhapper H Vitamin D status and its relation to age and body mass index Horm Res 2006 66 211 215 10.1159/000094932 16900001

[B10] Brock K Huang WY Fraser DR Ke L Tseng M Stolzenberg-Solomon R Peters U Ahn J Purdue M Mason RS McCarty C Ziegler RG Graubard B Low vitamin D status is associated with physical inactivity, obesity and low vitamin D intake in a large US sample of healthy middle-aged men and women J Steroid Biochem Mol Biol 2010 121 462 466 10.1016/j.jsbmb.2010.03.091 20399270 PMC2906665

[B11] Caron-Jobin M Morisset AS Tremblay A Huot C Légaré D Tchernof A Elevated serum 25(OH)D concentrations, vitamin D, and calcium intakes are associated with reduced adipocyte size in women Obesity (Silver Spring) 2011 19 1335 1341 10.1038/oby.2011.90 21527900

[B12] Chacko SA Song Y Manson JE Van Horn L Eaton C Martin LW McTiernan A Curb JD Wylie-Rosett J Phillips LS Plodkowski RA Liu S Serum 25-hydroxyvitamin D concentrations in relation to cardiometabolic risk factors and metabolic syndrome in postmenopausal women Am J Clin Nutr 2011 94 209 217 10.3945/ajcn.110.010272 21613558 PMC3127524

[B13] Cheng S Massaro JM Fox CS Larson MG Keyes MJ McCabe EL Robins SJ O’Donnell CJ Hoffmann U Jacques PF Booth SL Vasan RS Wolf M Wang TJ Adiposity, cardiometabolic risk, and vitamin D status: the Framingham Heart Study Diabetes 2010 59 242 248 10.2337/db09-1011 19833894 PMC2797928

[B14] Ding C Parameswaran V Blizzard L Burgess J Jones G Not a simple fat-soluble vitamin: changes in serum 25-(OH)D levels are predicted by adiposity and adipocytokines in older adults J Intern Med 2010 268 501 510 10.1111/j.1365-2796.2010.02267.x 20804516

[B15] Jacques PF Felson DT Tucker KL Mahnken B Wilson PW Rosenberg IH Rush D Plasma 25-hydroxyvitamin D and its determinants in an elderly population sample Am J Clin Nutr 1997 66 929 936 9322570 10.1093/ajcn/66.4.929

[B16] Jorde R Sneve M Emaus N Figenschau Y Grimnes G Cross-sectional and longitudinal relation between serum 25-hydroxyvitamin D and body mass index: the Tromsø study Eur J Nutr 2010 49 401 407 10.1007/s00394-010-0098-7 20204652

[B17] McCullough ML Weinstein SJ Freedman DM Helzlsouer K Flanders WD Koenig K Kolonel L Laden F Le Marchand L Purdue M Snyder K Stevens VL Stolzenberg-Solomon R Virtamo J Yang G Yu K Zheng W Albanes D Ashby J Bertrand K Cai H Chen Y Gallicchio L Giovannucci E Jacobs EJ Hankinson SE Hartge P Hartmuller V Harvey C Hayes RB Correlates of circulating 25-hydroxyvitamin D: Cohort Consortium Vitamin D Pooling Project of Rarer Cancers Am J Epidemiol 2010 172 21 35 10.1093/aje/kwq113 20562191 PMC2892536

[B18] Moschonis G Tanagra S Koutsikas K Nikolaidou A Androutsos O Manios Y Association between serum 25-hydroxyvitamin D levels and body composition in postmenopausal women: the Postmenopausal Health Study Menopause 2009 16 701 707 10.1097/gme.0b013e318199d5d5 19276997

[B19] Scragg R Camargo CA Frequency of leisure-time physical activity and serum 25-hydroxyvitamin D levels in the US population: results from the third National Health and Nutrition Examination Survey Am J Epidemiol 2008 168 577 586 10.1093/aje/kwn163 18579538 PMC2727193

[B20] Snijder MB van Dam RM Visser M Deeg DJ Dekker JM Bouter LM Seidell JC Lips P Adiposity in relation to vitamin D status and parathyroid hormone levels: a population-based study in older men and women J Clin Endocrinol Metab 2005 90 4119 4123 10.1210/jc.2005-0216 15855256

[B21] Zhou J Zhao LJ Watson P Zhang Q Lappe JM The effect of calcium and vitamin D supplementation on obesity in postmenopausal women: secondary analysis for a large-scale, placebo controlled, double-blind, 4-year longitudinal clinical trial Nutr Metab (Lond) 2010 7 62 10.1186/1743-7075-7-62 20650013 PMC3161354

[B22] Scragg R Holdaway I Jackson R Lim T Plasma 25-hydroxyvitamin D_3_ and its relation to physical activity and other heart disease risk factors in the general population Ann Epidemiol 1992 2 697 703 10.1016/1047-2797(92)90014-H 1342321

[B23] Van der Wielen RPJ Lowik MRH van den Berg H de Groot LC Haller J Moreiras O van Staveren WA Serum vitamin D concentrations among elderly people in Europe Lancet 1995 346 207 210 10.1016/S0140-6736(95)91266-5 7616799

[B24] Maetani M Maskarinec G Franke AA Cooney RV Association of leptin, 25-hydroxyvitamin D, and parathyroid hormone in women Nutr Cancer 2009 61 225 231 10.1080/01635580802455149 19235038 PMC4406327

[B25] McCarty MF Thomas CA PTH excess may promote weight gain by impeding catecholamine-induced lipolysis-implications for the impact of calcium, vitamin D, and alcohol on body weight Med Hypotheses 2003 61 535 542 10.1016/S0306-9877(03)00227-5 14592784

[B26] WHO (World Health Organization, ed.) Obesity. Preventing and managing the global epidemic 2000 Genf: WHO: WHO Technical Report Series 894 11234459

[B27] Roubenoff R Baumgartner RN Harris TB Dallal GE Hannan MT Economos CD Stauber PM Wilson PW Kiel DP Application of bioelectrical impedance analysis to elderly populations J Gerontol A Biol Sci Med Sci 1997 52 M129 M136 9158553 10.1093/gerona/52a.3.m129

[B28] Roche Diagnostics GmbH Elecsys 1010/2010/Modular analytics E170 2003 Mannheim Germany: PTH

[B29] Roche Diagnostics GmbH Elecsys and cobas analyzer 2007 Mannheim, Germany: Vitamin D3 (25-OH)

[B30] Holick MF Binkley NC Bischoff-Ferrari HA Gordon CM Hanley DA Heaney RP Murad MH Weaver CM Evaluation, treatment, and prevention of vitamin D deficiency: an Endocrine Society clinical practice guideline J Clin Endocrinol Metab 2011 96 1911 1930 10.1210/jc.2011-0385 21646368

[B31] Lührmann PM Herbert B Gaster C Neuhäuser-Berthold M Validation of a self-administered 3-day estimated dietary record for use in the elderly Eur J Nutr 1999 38 235 240 10.1007/s003940050066 10654160

[B32] Krems C Lührmann PM Neuhäuser-Berthold M Physical activity in young and elderly subjects J Sports Med Phys Fitness 2004 44 71 76 15181393

[B33] Lips P Vitamin D deficiency and secondary hyperparathyroidism in the elderly: consequences for bone loss and fractures and therapeutic implications Endocr Rev 2001 22 477 501 10.1210/er.22.4.477 11493580

[B34] Hintzpeter B Mensink GB Thierfelder W Müller MJ Scheidt-Nave C Vitamin D status and health correlates among German adults Eur J Clin Nutr 2008 62 1079 1089 10.1038/sj.ejcn.1602825 17538533

[B35] Orwoll E Nielson CM Marshall LM Lambert L Holton KF Hoffman AR Barrett-Connor E Shikany JM Dam T Cauley JA Vitamin D deficiency in older men J Clin Endocrinol Metab 2009 94 1214 1222 10.1210/jc.2008-1784 19174492 PMC2682464

[B36] Institute of Medicine (IoM) Ross AC, Taylor CL, Yaktine AL, Valle HB Committee to Review Dietary Reference Intakes for Vitamin D and Calcium Dietary reference intakes for calcium and vitamin D 2011 Washington DC: The National Academic Press 21796828

[B37] McGill AT Stewart JM Lithander FE Strik CM Poppitt SD Relationships of low serum vitamin D_3_ with anthropometry and markers of the metabolic syndrome and diabetes in overweight and obesity Nutr J 2008 7 4 10.1186/1475-2891-7-4 18226257 PMC2265738

[B38] Beydoun MA Boueiz A Shroff MR Beydoun HA Wang Y Zonderman AB Associations among 25-hydroxyvitamin D, diet quality, and metabolic disturbance differ by adiposity in adults in the United States J Clin Endocrinol Metab 2010 95 3814 3827 10.1210/jc.2010-0410 20463091 PMC2913037

[B39] Wortsman J Matsuoka LY Chen TC Lu Z Holick MF Decreased bioavailability of vitamin D in obesity Am J Clin Nutr 2000 72 690 693 10966885 10.1093/ajcn/72.3.690

[B40] Bolland MJ Grey AB Ames RW Horne AM Gamble GD Reid IR Fat mass is an important predictor of parathyroid hormone levels in postmenopausal women Bone 2006 38 317 321 10.1016/j.bone.2005.08.018 16199216

[B41] Kull M Kallikorm R Lember M Body mass index determines sunbathing habits: implications on vitamin D levels Intern Med J 2009 39 256 258 10.1111/j.1445-5994.2009.01900.x 19402866

[B42] Florez H Martinez R Chacra W Strickman-Stein N Levis S Outdoor exercise reduces the risk of hypovitaminosis D in the obese J Steroid Biochem Mol Biol 2007 103 679 681 10.1016/j.jsbmb.2006.12.032 17267209

[B43] Harris SS Dawson-Hughes B Reduced sun exposure does not explain the inverse association of 25-hydroxyvitamin D with percent body fat in older adults J Clin Endocrinol Metab 2007 92 3155 3157 10.1210/jc.2007-0722 17535990

[B44] Brot C Jorgensen NR Sørensen OH The influence of smoking on vitamin D status and calcium metabolism Eur J Clin Nutr 1999 53 920 926 10.1038/sj.ejcn.1600870 10602348

[B45] Ilich JZ Brownbill RA Tamborini L Crncevic-Orlic Z To drink or not to drink: how are alcohol, caffeine and past smoking related to bone mineral density in elderly women? J Am Coll Nutr 2002 21 536 544 12480799 10.1080/07315724.2002.10719252

[B46] Need AG Morris HA Horowitz M Nordin C Effects of skin thickness, age, body fat, and sunlight on serum 25-hydroxyvitamin D Am J Clin Nutr 1993 58 882 885 8249872 10.1093/ajcn/58.6.882

[B47] Binkley N Krueger DC Morgan S Wiebe D Current status of clinical 25-hydroxyvitamin D measurement: an assessment of between-laboratory agreement Clin Chim Acta 2010 411 1976 1982 10.1016/j.cca.2010.08.018 20713030 PMC3058672

[B48] Leino A Turpeinen U Koskinen P Automated measurement of 25-OH vitamin D_3_ on the Roche modular E170 analyzer Clin Chem 2008 54 2059 2062 10.1373/clinchem.2008.111732 18927245

[B49] Kyle UG Bosaeus I De Lorenzo AD Deurenberg P Elia M Manuel Gómez J Lilienthal Heitmann B Kent-Smith L Melchior JC Pirlich M Scharfetter H M W J Schols A Pichard C Bioelectrical impedance analysis-part II: utilization in clinical practice Clin Nutr 2004 23 1430 1453 10.1016/j.clnu.2004.09.012 15556267

